# Containment of Highly Pathogenic Avian Influenza A(H5N1) Virus, Lebanon, 2016^1^

**DOI:** 10.3201/eid2402.171276

**Published:** 2018-02

**Authors:** Zeina E. Farah, Omaya Khatib, Sahar Hamadeh, Khadija Ahmad, Bassel El Bazzal, Pierre Zalloua, Walid Ammar, Nada Ghosn

**Affiliations:** Ministry of Public Health, Bekaa, Lebanon (Z.E. Farah, O. Khatib, S. Hamadeh, K. Ahmad);; Ministry of Agriculture, Beirut, Lebanon (B. El Bazzal);; National Influenza Center, Beirut (P. Zalloua);; Ministry of Public Health, Beirut (W. Ammar, N. Ghosn)

**Keywords:** influenza, influenza virus, viruses, highly pathogenic avian influenza virus, H5N1 subtype, poultry, humans, antiviral drugs, respiratory infections, containment, Lebanon

## Abstract

A preparedness plan for avian influenza A(H5N1) virus infection was activated in Lebanon in 2016 after reported cases in poultry. Exposed persons were given prophylaxis and monitored daily. A total of 185 exposed persons were identified: 180 received prophylaxis, 181 were monitored, and 41 suspected cases were reported. All collected specimens were negative for virus by PCR.

Highly pathogenic avian influenza A(H5N1) virus has caused ≈1,000 human infections since the first case was reported in 1997 (overall case-fatality rate 54%) (*1*). The number of affected countries increased during 2003−2008, and infections spread from East and Southeast Asia to West Asia and several regions of Africa (*2*). The highest cumulative number of confirmed human cases was reported in Egypt (*3**,**4*). This virus has been detected in poultry and wild birds in >50 countries, and the virus is epizootic in Bangladesh, China, Egypt, India, Indonesia, and Vietnam (*5*).

Since 2006, several infections with this virus have been detected in areas in the Middle East, including Iran, Jordan, Kuwait, Saudi Arabia, Turkey, the West Bank, the Gaza Strip, and Yemen. In 2015, outbreaks of infection with this virus were reported in the regions of the Gaza Strip, the West Bank, Turkey, and Iran (*6*). In addition, 6 outbreaks of infection with this virus were reported in Iraq during December 2015−February 2016 (*6*). Although no cases have been reported in Syria, the Food and Agriculture Organization of the United Nations is concerned about circulation of this virus because of extensive poultry production in Syria and the low biosecurity along its border with Iraq (*6*).

In Lebanon, no cases of infection with avian influenza A(H5N1) virus were reported in poultry or humans before 2016. On the basis of World Health Organization recommendations, a multisectoral preparedness plan was developed in 2007. The purpose of the plan was early detection and containment of any influenza outbreak caused by this virus and to ensure good coordination between public health sectors and ministries (agriculture, health, interior, and environment), the national influenza center, and laboratories (*7*).

## The Study

On April 20, 2016, the Lebanonese Ministry of Agriculture confirmed the presence of avian influenza A(H5N1) virus on 2 poultry farms in Nabi Chit village located in the Bekaa region, adjacent to the border of Lebanon with Syria. This virus caused the deaths of 20,000 domestic birds. Consequently, the preparedness plan was activated after a multisectoral meeting attended by representatives from the Ministry of Agriculture, the Ministry of Public Health, the Ministry of Interior, the Lebanese Army, and the local community.

The Ministry of Agriculture culled all domestic birds within a 3-km radius of infected farms. A total of 60,000 birds were culled from 7 farms (including the 2 infected farms) and 10 households with backyards. All farms were disinfected, and organic remains were disposed safely. Indemnity was provided to farmers by the Higher Relief Council. Epidemiologic investigations indicated that illegal movement of animals was the possible source of infections. The isolated virus (clade 2.3.2.1c) was similar to that detected in wild and domestic birds in Bulgaria, Romania, and Turkey during January−March 2015 (*6*).

The Ministry of Public Health plan focused on exposure identification, early detection of cases, and awareness. Identification of all exposed persons was crucial for providing prophylaxis and early detection of any human case. An exposed person was defined as any person who was exposed to 1) poultry or their remains or 2) environments contaminated by poultry feces in the area targeted by the Ministry of Agriculture, regardless of the use of personal protective equipment. Lists of target farms and households, as well as field teams working in culling and disinfection, were provided by the Ministry of Agriculture. A field team from the Ministry of Public Health visited the target farms and households and identified exposed persons. Exposed persons were also identified in 2 assigned health centers in the village and neighborhood. The laboratory team that handled infected specimens and the veterinarian who examined infected poultry were also considered to be exposed persons.

Oseltamivir was used as prophylaxis and orally administered to persons >1 year of age up to 10 days after the last documented exposure (75 mg/d for adults and 35 mg/d for children). Exposed persons were monitored on a daily basis by telephone calls to assess their clinical status and to check for respiratory symptoms. For persons with signs or symptoms, oropharyngeal swab specimens were collected and tested at the National Influenza Center by using real-time reverse transcription PCR.

Three sensitization sessions for health professionals working in hospitals, medical centers, and private clinics were conducted to enhance detection and reporting of any suspected case of infection with influenza A(H5N1) virus. We adopted a highly sensitive H5N1 subtype case-patient definition. We defined a suspected case-patient as an exposed person with fever (temperature >38°C) or respiratory signs/symptoms (cough, dyspnea, coryza, sore throat) after April 20, 2016, who lived or worked in Nabi Chit village. In addition, 3 health education sessions were conducted for the public in collaboration with the local community to increase awareness about avian influenza.

We entered data into Epidata version 3.1 software (http://www.epidata.dk/download.php). We then analyzed data by using EpiDataStat version 2.2.1.171 software (http://www.advanceduninstaller.com/EpiData-Analysis-2_2_1_171-fb08f19a7f340dae85e8b2d85e90c006-application.htm).

We identified 185 exposed persons; 138 were male, 96 were Lebanese, and 88 were Syrian (mean age 30 y) (Table 1). A total of 151 had contact with poultry on farms or near their homes, and 34 were members of response teams. Of 185 exposed persons, 180 (97.3%) received antiviral prophylaxis (86.1% received the adult dose, and 13.9% received the pediatric dose), and 5 did not receive antivirus prophylaxis (4 were <1 year of age, and 1 refused prophylaxis). Duration of antivirus therapy ranged from 10 to 30 days. Of 185 exposed persons, 183 (99.0%) were monitored for >7 days, 1 was monitored for 4 days and then was lost to follow up, and 1 refused to participate. A total of 41 persons who received prophylaxis had signs/symptoms. The most commonly reported symptom was coryza (65.9%) (Table 2). Specimens were collected from 39 persons. In addition, 2 hospitalized suspected case-patients were tested. All results were negative for H5N1 subtype virus by real-time reverse transcription PCR.

**Table 1:** Demographic chacteristics of 185 persons exposed to highly pathogenic avian influenza A(H5N1) virus, Lebanon, 2016*

Characteristic	Value
Sex	
M	138 (74.6)
F	47 (25.4)
Mean age, y (range)	29.8 (2 mo−89 y)
Nationality	
Lebanese	96 (51.9)
Syrian	88 (47.6)
Ethiopian	1 (0.5)
Exposure	
Farms	78 (42.2)
Households	41 (22.2)
Neighborhood	32 (17.3)
Municipality team	18 (9.7)
Ministry of Agriculture team	14 (7.6)
Laboratory team	2 (1.1)

*Values are no. (%) unless otherwise indicated.

**Table 2:** Reported signs/symptoms of 41 persons with suspected cases of infection with highly pathogenic avian influenza A(H5N1) virus, Lebanon, 2016

Sign/symptom	No. (%)
Coryza	27 (65.9)
Cough	24 (58.5)
Sore throat	18 (43.9)
Dyspnea	11 (26.8)
Fever	7 (17.1)

On May 10, 2016, twenty days after the start of the outbreak in the first village, another focus appeared in the neighboring village of Sariin Tehta. We used the same case definition for suspected cases and detected 8 suspected case-patients; specimens were collected from 7, but no H5N1 cases were detected. The association between the 2 foci remains unclear. Since then, repetitive sampling conducted by the Ministry of Agriculture did not detect any infections in poultry. The 2 outbreaks were reported to the World Health Organization and the World Organisation for Animal Health within 24 h of confirmation (*8*), and containment was declared in June 2016.

## Conclusions

Infections with avian influenza A(H5N1) virus in Lebanon in 2016 resulted in the deaths of 20,000 poultry and culling of 60,000 poultry. Although 185 persons were exposed to this virus, no human cases were identified. The reason behind the successful containment of the infection foci was early intervention of the Ministries of Agriculture and Public Health and use of a preparedness plan. This proactive effort enabled efficient coordination in the context of an acute shortage of human resources and rapid dissemination of false information about avian influenza. Because of high risk for new infections with this virus, the next step is to update the plan on the basis of lessons learned during the recent outbreaks.
