# Correction: Vol. 24, No. 2

**DOI:** 10.3201/eid2406.C42406

**Published:** 2018-06

**Keywords:** errata, erratum, correction

The affiliation of author Pierre Zalloua was listed incorrectly in Containment of Highly Pathogenic Avian Influenza A(H5N1) Virus, Lebanon, 2016 (Z.E. Farah et al.). He is affiliated with Lebanese American University. The article has been corrected online (https://wwwnc.cdc.gov/eid/article/24/2/17-1276_article).

